# Apa-I polymorphism in *VDR* gene is related to metabolic syndrome in polycystic ovary syndrome: a cross-sectional study

**DOI:** 10.1186/s12958-018-0355-9

**Published:** 2018-04-18

**Authors:** Betânia Rodrigues Santos, Sheila Bunecker Lecke, Poli Mara Spritzer

**Affiliations:** 1 Division of Endocrinology, Gynecological Endocrinology Unit, Hospital de Clínicas de Porto Alegre, Rua Ramiro Barcelos, 2350, Porto Alegre, RS 90035-003 Brazil; 2 Department of Physiology, Laboratory of Molecular Endocrinology, Universidade Federal do Rio Grande do Sul (UFRGS), Porto Alegre, Brazil; 3 Department of Diagnostic Methods, Universidade Federal de Ciências Médicas de Porto Alegre (UFCSPA), Porto Alegre, Brazil

**Keywords:** PCOS, Vitamin D receptor, Gene polymorphisms, Metabolic syndrome

## Abstract

**Background:**

Polycystic ovary syndrome (PCOS) is a common endocrine disorder determined by polygenic traits as well as environmental factors. Lower vitamin D levels have been detected in PCOS women and related to hormone and metabolic disturbances. Vitamin D acts in tissues through the vitamin D receptor (VDR). *VDR* gene variants have been associated with worse metabolic profile in the general population. We investigated the genotype and haplotype distribution of the Bsm-I (rs1544410), Apa-I (rs7975232), and Taq-I (rs731236) *VDR* gene polymorphisms in PCOS and non-hirsute women from southern Brazil. We further investigated the associations of these gene variants and their haplotypes with PCOS, vitamin D levels, and metabolic abnormalities, including the metabolic syndrome (MetS).

**Methods:**

A group of 191 women with PCOS (Rotterdam criteria) and 100 non-hirsute controls with regular ovulatory cycles were genotyped for all polymorphisms by real-time PCR, with allelic discrimination assays. MetS and the cutoffs for its isolated components were defined in accordance with the Joint Scientific Statement.

**Results:**

Women with PCOS were younger and had significantly higher BMI and total testosterone levels than controls (*p* < 0.05). The frequency of MetS in PCOS and controls was 26.5% and 4.8% respectively. The CC genotype of Apa-I entailed higher risk of MetS in PCOS (OR: 2.133; 95% CI 1.020–4.464, *p* = 0.042), and was associated with higher systolic blood pressure (*p* = 0.009), total cholesterol (*p* = 0.040), and LDL-cholesterol (*p* = 0.038) in both PCOS and control groups (two-way ANOVA). The frequencies of *VDR* haplotypes were similar in PCOS and control women.

**Conclusions:**

The present results suggest that the Apa-I variant in *VDR* gene may be associated with MetS in southern Brazilian women with PCOS, and with blood pressure, total cholesterol, and LDL-c in women with and without PCOS.

## Background

Polycystic ovary syndrome (PCOS) is a common endocrine disorder affecting 9 to 18% of women of reproductive age according to different diagnostic criteria [1–3]. While its etiology remains unclear, PCOS is considered a polygenic and multifactorial disease, with metabolic, endocrine, and reproductive alterations [4]. In PCOS women, evidence suggests that vitamin D levels may be decreased and related to hormone and metabolic disturbances [5, 6].

The vitamin D receptor (VDR) is expressed in many tissues and organs (such as those involved in calcium homeostasis mechanisms), in glucose metabolism, and in the reproductive system [7], and modulates vitamin D action in these systems. *VDR* gene (ID: 7421) polymorphisms have been investigated in PCOS as well as in disturbances of androgen secretion. A previous study has suggested an association between *VDR* gene variants and precocious pubarche (PP) [8]; in turn, data on PCOS risk are controversial, with a relationship between *VDR* gene variants and PCOS detected by some [9–12] but not all studies [13–15]. Regarding endocrine characteristics, *VDR* gene polymorphism has been associated with total testosterone in PCOS and PP populations [8, 13], with estradiol levels in PP girls [8], and with metabolic abnormalities in different non-PCOS populations [16–23].

Therefore, the aims of the present study were to assess the genotypic and allelic distribution of Bsm-I (rs1544410), Apa-I (rs7975232) and Taq-I (rs731236) polymorphisms of the *VDR* gene and to determine whether these gene variants are associated with 25-hydroxyvitamin D [25(OH)D] levels and with metabolic abnormalities, including MetS, in women with PCOS in comparison to non-hirsute, ovulatory control women.

## Methods

### Patients

This is a cross-sectional study including 191 patients with PCOS and 100 non-hirsute women with regular, ovulatory cycles, recruited by advertisement in the local media. The characteristics of the study sample have been described elsewhere [24]. PCOS was diagnosed according to Rotterdam criteria [25]. Neither PCOS nor control participants had received any drugs known to interfere with hormone levels (such as oral contraceptive pills, antiandrogens, metformin, fibrates, or statins) for at least 3 months before the study. The exclusion criteria were pregnancy and liver or kidney disease. Approval for this study was obtained from the Institutional Review Board and the local Ethics Committee at Hospital de Clínicas de Porto Alegre. Written informed consent was obtained from every subject.

### Study protocol

Anthropometric measurements included body mass index (BMI) and waist circumference (measured at the midpoint between the lower rib margin and the iliac crest). Blood pressure was measured after a 10-min rest, with the patient seated, with both feet on the floor and the arm supported at heart level. Two measurements were obtained 10 min apart using an Omron HEM-742INT automatic blood pressure monitor (Rio de Janeiro, Brazil) with the correct cuff size for the arm diameter [26–29]. MetS and the cutoffs for its isolated components were defined in accordance with the Joint Scientific Statement [30].

### Laboratory measurements

All samples were obtained between the 2nd and 10th days of the menstrual cycle, or on any day if the patient was amenorrheic, between 8:00 and 10:00 am, after a 12-h overnight fast. Blood samples were drawn from an antecubital vein for determination of hormone levels**.** Blood samples were also collected for genomic DNA extraction.

Total cholesterol, high-density lipoprotein cholesterol (HDL-c), triglycerides, and glucose levels were determined by colorimetric-enzymatic methods (Bayer 1650 Advia System). LDL-cholesterol (LDL-c) was determined indirectly with the formula total cholesterol – HDL-c – triglycerides/5. Total testosterone levels were measured by chemiluminescence (Siemens Advia Centaur XP), with a sensitivity of 0.10 ng/mL and intra- and interassay coefficients of variation (CVs) of 3.3 and 7.5% respectively. Plasma insulin and sex hormone–binding globulin (SHBG) levels were measured by chemiluminescence (Siemens Advia Centaur XP), with a sensitivity of 0.50 U/mL and 0.035 nmol/L, respectively, with intra-assay CV < 3% and interassay CV < 5%. The free androgen index (FAI) was calculated as testosterone (nmol/L)/SHBG (nmol/L) × 100. The homeostasis model assessment index (HOMA index) was calculated by multiplying insulin (μIU/mL) by glucose (mmol/L) and dividing this product by 22.5 [31]. 25(OH)D levels were measured in a subset of 102 women (54 PCOS and 48 controls) by chemiluminescence (Liaison, DiaSorin), with intra-assay and interassay CV of 7.7 and 10.9% respectively.

### Genotype analysis

Genomic DNA was extracted from peripheral blood leukocytes [32]. The DNA samples were diluted to 2 ng/mL. Molecular genotyping was performed through real-time polymerase chain reaction (7500 Fast Real-Time Polymerase Chain Reaction System, Applied Biosystems, CA, USA), using the allelic discrimination assay with TaqMan MGB primers and probes (Applied Biosystems, CA, USA).

For genotyping the single nucleotide polymorphisms (SNPs) Apa-I and Taq-I, the following were added: TaqMan Master mix (2.5 μL), TaqMan assay (0.25 μL), and H_2_O (1.25 μL), for a final volume of 4 μL per sample, followed by addition of 1μLof DNA for a total reaction volume of 5 μL. To genotype SNP Bsm-I, TaqMan Master mix (5.0 μL), TaqMan assay (0.50 μL), and H_2_O (3.5 μL) were added for a final volume of 9 μL per sample, and 1 μL of DNA was added for a total reaction volume of 10 μL. Reaction conditions for all polymorphisms were: 10 min at 95 °C after 50 cycles of denaturation at 95 °C (15 s) and annealing at 60 °C (1 min). Endpoint fluorescent readings were performed in the 7500 Fast System Sequence Detection Software version 1.4 environment. The internal quality of genotype data was assessed by typing 10% of blinded samples in duplicate.

### Statistical analysis

Sample size estimation was based on the study by Al-Daghri et al. [16], which found an association between Apa-I variants of the *VDR* gene and higher blood pressure in MetS in female and male control subjects. Therefore, considering a difference of 3.9 mmHg in blood pressure between Apa-I genotypes [CC or CA + AA], an alpha of 5%, and a beta of 80%, the sample size was estimated as 91 PCOS women for each genotype.

The Shapiro-Wilk normality test and descriptive statistics were used to evaluate the distribution of data. Results are presented as means ± standard deviation or percentages. Non-Gaussian variables were log-transformed for statistical analysis and reported after being back-transformed into their original units of measure. Comparisons between means were analyzed by the unpaired two-tailed Student’s t-test. Two-way ANOVA was used for testing the interaction between diagnosis and genotype groups. Categorical variables and the agreement of genotype frequencies with Hardy-Weinberg equilibrium for each SNP were analyzed using the Pearson chi-square test (χ^2^). Odds ratios (OR) and 95% confidence intervals (95%CI) were obtained using χ^2^ risk estimate. Lewontin’s D’ statistic for linkage disequilibrium was calculated for each pair of polymorphisms. Haplotypes were inferred using the Phase 2.1 program, which uses Bayesian statistics. Data were considered as statistically significant at *p* < 0.05. The Statistical Package for the Social Sciences v. 24 (SPSS, Chicago, IL) was used for the analyses.

## Results

### Clinical, hormonal, and metabolic features

The clinical, hormonal and metabolic characteristics of the studied population have been previously described [24]. Women with PCOS were younger than controls (22.9 ± 6.7 vs. 25.2 ± 7.7 years, *p* = 0.013), and presented higher BMI (29.7 ± 6.4 vs. 27.0 ± 6.1 kg/m^2^, *p* = 0.001) and higher frequency of overweight/obesity (*p* = 0.002) and of MetS (*p* < 0.001). PCOS participants also had significantly higher total testosterone and FAI, as well as lower SHBG (p < 0.001), than controls (Table 1). Vitamin D levels were similar in PCOS and controls (*p* = 0.985).

**Table 1:** Clinical and endocrine features of PCOS and control women

Variable	PCOS (191)	Controls (100)	*P* value
Age (years)	22.89 ± 6.66	25.18 ± 7.72	0.013
BMI ≥ 25(kg/m^2^)	72.6%	53.4%	0.002
Metabolic syndrome	26.5%	4.8%	< 0.001
TT (ng/mL)	0.90 ± 0.41	0.54 ± 0.17	< 0.001
FAI	16.52 ± 15.81	5.28 ± 3.41	< 0.001
SHBG (nmol/L)	29.18 ± 20.35	43.37 ± 19.37	< 0.001
25(OH)D (ng/mL)	21.47 ± 7.61	21.50 ± 6.90	0.985

Data are expressed as means ± SD (Student t test) or percentages (Pearson chi-square test). *BMI* body mass index, *TT* total testosterone, *FAI* free androgen index, *SHBG* sex hormone–binding globulin, *25(OH)D* 25-hydroxyvitamin D

When only the PCOS group was analyzed, the presence of MetS (*p* = 0.018), glucose ≥100 mg/dL (*p* = 0.025), waist circumference ≥ 88 cm (*p* = 0.040) and triglycerides ≥150 mg/dL (*p* = 0.011) were linked to lower vitamin D levels (Table 2).

**Table 2:** 25(OH)D levels according to the presence of metabolic syndrome and its components in PCOS women

	25(OH)D levels (ng/mL)					
Status of MetS/components	MetS	Glu ≥100 mg/dL	BP ≥130/85 mmHg	WC ≥88 cm	HDL-c < 50 mg/dL	Trig ≥150 mg/dL
Yes	17.17 ± 5.46	14.83 ± 6.24	23.25 ± 7.80	19.28 ± 5.92	21.50 ± 7.66	17.84 ± 4.37
No	22.83 ± 7.74	22.22 ± 7.47	20.78 ± 7.61	23.46 ± 8.47	21.42 ± 7.74	22.71 ± 7.85
p value	0.018	0.025	0.318	0.040	0.974	0.011

Data are expressed as means ± SD. P value by Student t test. Glu: glucose; BP: blood pressure; WC: waist circumference; HDL-c: high-density lipoprotein cholesterol. Trig: triglycerides

### *VDR* gene polymorphisms

All the three studied polymorphisms were in Hardy-Weinberg equilibrium, and over 98% (*n* = 287) of the sample were effectively genotyped. Genotype and allele frequencies of *VDR* gene variants are presented in Table 3. The genotype and allele distribution of all three polymorphisms was similar in PCOS and control groups.

**Table 3:** Genotype and allele frequencies of *VDR* gene variants in PCOS and control women

SNP	PCOS n (%)	Controls n (%)	p
Bsm-I			
GG	74 (39.6)	41 (41.0)	0.147
GA	76 (40.6)	48 (48.0)	
AA	37 (19.8)	11 (11.0)	
G	224 (60.0)	130 (65.0)	0.231
A	150 (40.0)	70 (35.0)	
Apa-I			
AA	61 (32.1)	36 (36.0)	0.516
AC	88 (46.3)	48 (48.0)	
CC	41 (21.6)	16 (16.0)	
A	210 (55.3)	120 (60.0)	0.275
C	170 (44.7)	80 (40.0)	
Taq-I			
AA	70 (37.2)	40 (40.4)	0.493
AG	87 (46.3)	48 (48.5)	
GG	31 (16.5)	11 (11.1)	
A	227 (60.4)	128 (64.6)	0.318
G	149 (39.6)	70 (35.4)	

Data are expressed as percentages; p value by Pearson’s χ^2^ test

**Fig. 1:**
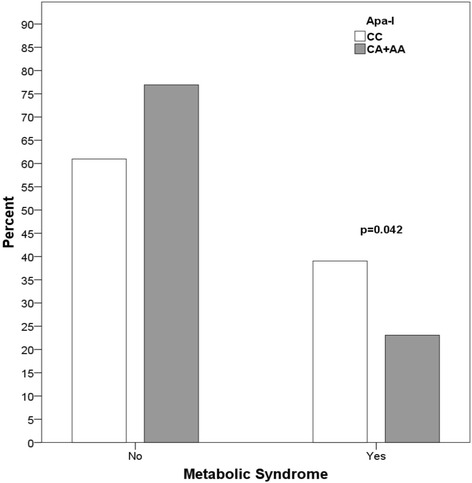
Frequency of metabolic syndrome in PCOS women according to Apa-I genotypes. Data are expressed as percentages (Pearson chi-square test). Frequency values: Apa-I: No – CC: 61.0%; CA + AA: 76.9% / Yes – CC: 39.0%; CA + AA: 23.1%. OR: 2.133; 95% CI: 1.020–4.464

Figure 1 shows the frequency of MetS in PCOS participants according to Apa-I genotypes. Individuals with the CC genotype had higher risk of MetS vs. the CA + AA genotype (OR: 2.133; 95% CI 1.020–4.464, *p* = 0.042). The CC genotype was also associated with higher systolic blood pressure (*p* = 0.009), total cholesterol (*p* = 0.040) and LDL-c (*p* = 0.038) in both PCOS and control groups. There was no interaction between genotypes and PCOS or control groups (*p* > 0.05) (Table 4).

**Table 4:** Clinical, endocrine, and metabolic features of PCOS and control women according to presence or absence of Apa-I SNP

Variable	PCOS (*n* = 190)		Controls (*n* = 100)		
	CC (41)	CA + AA (149)	CC (16)	CA + AA (84)	p _gen_
WC (cm)^#^	91.97 ± 15.45	88.56 ± 14.96	81.42 ± 13.08	77.41 ± 11.20	0.149
SBP (mmHg)^#^	127.02 ± 19.79 ^a^	119.49 ± 13.69 ^b^	113.83 ± 11.35 ^a^	108.60 ± 13.10 ^b^	0.009
DBP (mmHg)^#^	81.30 ± 13.46	77.21 ± 10.84	72.77 ± 10.17	70.41 ± 9.24	0.079
Glucose (mg/dL)	87.88 ± 13.18	89.14 ± 15.92	90.47 ± 7.13	88.08 ± 7.65	0.805
Insulin (μUI/mL)^#^	20.28 ± 12.85	22.41 ± 21.27	11.64 ± 5.78	12.03 ± 6.71	0.846
HOMA-IR^#^	4.45 ± 3.07	5.12 ± 5.86	2.56 ± 1.45	2.50 ± 1.60	0.654
TC (mg/dL)	180.95 ± 37.35 ^a^	172.73 ± 38.55 ^b^	183.80 ± 36.87 ^a^	167.26 ± 28.76 ^b^	0.040
HDL-c (mg/dL)^#^	46.12 ± 10.73	49.59 ± 10.86	53.73 ± 11.21	52.65 ± 12.55	0.529
LDL-c (mg/dL)	111.05 ± 31.97 ^a^	102.52 ± 31.69 ^b^	111.99 ± 32.73 ^a^	99.56 ± 24.48 ^b^	0.038
Trig (mg/dL)^#^	118.90 ± 99.18	104.29 ± 62.18	90.40 ± 55.15	75.28 ± 41.89	0.149
25(OH)D (ng/mL)	19.41 ± 5.23	22.12 ± 8.17	21.31 ± 6.15	21.52 ± 7.16	0.399

*WC* waist circumference, *SBP* systolic blood pressure, *DBP* diastolic blood pressure, *HOMA* homeostasis model assessment index, *TC* total cholesterol, *HDL-c* high-density lipoprotein cholesterol, *LDL-c* low-density lipoprotein cholesterol, *Trig* triglycerides, 25(OH)D 25-hydroxyvitamin D

Values are expressed as means ± SD (two-way ANOVA). Different superscript letters indicate statistical difference for comparisons between genotypes, grouped by the absence or presence of the polymorphic allele, in PCOS and control groups. ^#^ *p* < 0.005 for comparisons between PCOS (CC and CA + AA) and control (CC and CA + AA) groups

The Bsm-I (G → A) polymorphism was in almost complete linkage disequilibrium with the Apa-I (C → A) polymorphism (|D’| = 1.00; *r*^2^ = 1.00), and in partial linkage disequilibrium with Taq-I (A → G) (|D’| = 0.75; *r*^2^ = 0.21). Apa-I (C → A) was also in partial linkage disequilibrium with Taq-I (A → G) (|D’| = 0.87; *r*^2^ = 0.35). Eight haplotypes were inferred in the sample: AAA, AAG, ACA, ACG, GAA, GAG, GCA, and GCG, with frequencies of 0.022, 0.340, 0.015, 0.004, 0.192, 0.019, 0.393, and 0.015 respectively. The first letter of each haplotype refers to Bsm-I, the second to Apa-I, and the third to Taq-I. Taking into consideration the results of individual polymorphism analyses, haplotypes were grouped according to the presence of the C allele of Apa-I (ACA + ACG + GCA + GCG vs. AAA + AAG + GAA + GAG). The frequency of combined haplotypes was similar in PCOS and control groups (*p* = 0.332).

## Discussion

In the present study, despite the similar vitamin D levels detected in PCOS and control participants, the CC genotype of Apa-I SNP of the *VDR* gene was specifically related to higher risk of MetS in PCOS participants. Moreover, this same genotype was associated with higher blood pressure, total cholesterol, and LDL-c in both PCOS and control participants. To the best of our knowledge, this is the first report to show an association between Apa-I *VDR* gene polymorphism and MetS in a PCOS population. This observation is relevant because it may help explain the meaning of vitamin D level variation, which may not play a role per se, but rather reflect a putative gene-environment interaction in different populations.

The few available studies analyzing the influence of Apa-I *VDR* gene polymorphisms on metabolic variables in PCOS women have reported no association with insulin resistance [10, 13] or glucose and lipid abnormalities [10]. However, data from non-PCOS populations suggest that metabolic abnormalities, such as obesity, insulin resistance, low HDL-c, and type 2 diabetes are associated with the *VDR* gene [16–21]. In this sense, a recent meta-analysis comprising 9232 participants showed that the association between insulin resistance-related diseases and Apa-I and Bsm-I *VDR* gene variants was more pronounced in dark-pigmented Caucasians and Asians than in Caucasians with white skin. In the sub-group analysis, Bsm-I (GG genotype) was associated with MetS, and the Apa-I variant (CC genotype) was associated with insulin resistance-related diseases in a population living in a mid-latitude zone (30°–60°) [23], which is also the case of the present population (30°01′59”S).

While a functional role of *VDR* gene polymorphisms has not yet been established, the association between Apa-I gene variant and MetS observed in the present study could be assumed to be linked to disturbed *VDR* gene expression [33]. The Apa-I polymorphism is located at the 3′ untranslated region (3′ UTR) of the *VDR* gene, which has been recognized as being involved in the modulation of gene expression, especially through the regulation of mRNA stability and efficiency of protein translation [34]. Moreover, the methylation levels of the *VDR* gene appear to be altered according to race and presence of the polymorphisms of the 3’UTR region of the gene [35]. Additionally, Apa-I is in strong linkage disequilibrium with other *VDR* gene polymorphisms in different populations [22, 36], which may be contributing to the general transcriptional activity of *VDR* in different biological processes. Importantly, the *VDR* gene regulates more than 200 genes, and mediates most effects of vitamin D on gene expression via formation of a heterodimer with the retinoid X receptor molecule, which binds to promoter regions of many target genes [37, 38].

In our study, lower 25(OH)D levels were associated with MetS and with its isolated components in PCOS women, such as higher glucose, waist circumference and triglycerides. In this sense, the present results are in agreement with a meta-analysis reporting that women with PCOS and vitamin D deficiency are more likely to have dysglycemia compared to those without vitamin D deficiency [5], and that in women with both PCOS and MetS, vitamin D levels are lower than in women with PCOS and without MetS [39].

Similar vitamin D levels were detected in the present study in PCOS and control participants regardless of the presence of Apa-I SNP. Interestingly, while two meta-analyses [5, 6] comprising 3182 and 2262 women respectively showed that serum 25(OH)D concentrations were lower in PCOS compared to controls, the reported standardized mean difference between the groups in both studies seems of little clinical relevance – only 0.74 ng/mL (95%IC: -1.26 to − 0.22) [5] and 0.64 ng/mL (95%IC: -1.12 to − 0.15) [6]. In turn, the fact that our PCOS patients with MetS had lower vitamin D levels and higher frequency of CC polymorphism compared to those without MetS suggests that the Apa-I gene variant might impact vitamin D levels in PCOS with MetS. In fact, vitamin D status is influenced by many factors, especially dietary pattern, season, and genetic traits [40]. A better understanding of the genetic factors that may be involved in vitamin D level variation and metabolic disturbances could shed some light on hypothetical gene-environment interactions of vitamin D. Further studies with larger PCOS populations and higher proportion of MetS are needed in order to confirm this hypothesis.

We did not find any association between genotypes or haplotypes of *VDR* gene variants in PCOS participants. Only a few studies are available in the literature assessing *VDR* gene polymorphism and risk of PCOS, with uncertain conclusions, which vary according to the studied sample. While some studies show an association between at least one *VDR* gene polymorphism and PCOS [9–12, 41], others report similar distributions of Bsm-I, Apa-I and Taq-I polymorphisms in PCOS and control women [13–15]. Also, regarding haplotypes of *VDR* gene variants, no definitive data are available, with few reports of distinct haplotypes of *VDR* gene polymorphisms presenting slightly higher frequency in PCOS women when compared to controls [10, 12, 14]. These unclear data may be, at least in part, attributed to ethnic differences in the studied populations and to the polygenic condition of PCOS. Yet, other studies have reported an association of *VDR* gene polymorphisms with PP [8] and diabetes [42–45].

One strength of our study is the focus on a less well represented ethnic group, PCOS women from southern Brazil, with assessment of gene variants which may be contributing to this polygenic and multifactorial disease. Furthermore, we evaluated polymorphisms found in a genomic position that plays an important role in the modulation of gene expression. Limitations of the present study are the relatively small sample size of 291 participants (191 PCOS and 100 controls) and the low frequency of MetS in the control group, precluding complementary analyses correlating *VDR* gene polymorphisms and MetS in that group. In addition, further studies on functional evaluation of *VDR* SNPs are needed in order to deepen the understanding of findings.

## Conclusions

Our results indicate that Bsm-I, Apa-I, and Taq-I polymorphisms in *VDR* gene are not related to PCOS. However, there seems to be an association of the CC genotype of Apa-I with MetS in PCOS women, and with blood pressure, total cholesterol, and LDL-c in women with and without PCOS. Despite the similarity in the vitamin D levels of PCOS and control participants, our study suggests that Apa-I impacts vitamin D levels in PCOS with MetS.

## Abbreviations

25(OH)D: 25-hydroxyvitamin D
BMI: Body mass index
BP: Blood pressure
CV: Coefficient of variation
DBP: Diastolic blood pressure
FAI: Free androgen index
Glu: Glucose
HDL-c: High-density lipoprotein cholesterol
HOMA: Homeostasis model assessment index
LDL-c: Low-density lipoprotein cholesterol
MetS: Metabolic syndrome
PCOS: Polycystic ovary syndrome
PCR: Polymerase chain reaction
PP: Precocious pubarche
SBP: Systolic blood pressure
SHBG: Sex hormone–binding globulin
SNP: Single nucleotide polymorphism
TC: Total cholesterol
Trig: Triglyceride
TT: Total testosterone
VDR: Vitamin D receptor
WC: Waist circumference

## References

[1] Azziz R, Woods KS, Reyna R, Key TJ, Knochenhauer ES, Yildiz BO (2004). The prevalence and features of the polycystic ovary syndrome in an unselected population. J Clin Endocrinol Metab.

[2] Asuncion M, Calvo RM, San Millan JL, Sancho J, Avila S, Escobar-Morreale HF (2000). A prospective study of the prevalence of the polycystic ovary syndrome in unselected Caucasian women from Spain. J Clin Endocrinol Metab.

[3] March WA, Moore VM, Willson KJ, Phillips DIW, Norman RJ, Davies MJ (2010). The prevalence of polycystic ovary syndrome in a community sample assessed under contrasting diagnostic criteria. Hum Reprod.

[4] De Leo V, Musacchio MC, Cappelli V, Massaro MG, Morgante G, Petraglia F (2016). Genetic, hormonal and metabolic aspects of PCOS: an update. Reprod Biol Endocrinol.

[5] He CL, Lin ZM, Robb SW, Ezeamama AE (2015). Serum vitamin D levels and polycystic ovary syndrome: a systematic review and meta-analysis. Nutrients.

[6] Bacopoulou F, Kolias E, Efthymiou V, Antonopoulos CN, Charmandari E (2017). Vitamin D predictors in polycystic ovary syndrome: a meta-analysis. Eur J Clin Investig.

[7] Palomer X, Gonzalez-Clemente JM, Blanco-Vaca F, Mauricio D (2008). Role of vitamin D in the pathogenesis of type 2 diabetes mellitus. Diabetes Obes Metab.

[8] Santos BR, Mascarenhas LP, Satler F, Boguszewski MC, Spritzer PM (2012). Vitamin D receptor gene polymorphisms and sex steroid secretion in girls with precocious pubarche in southern Brazil: a pilot study. J Endocrinol Investig.

[9] Mahmoudi T (2009). Genetic variation in the vitamin D receptor and polycystic ovary syndrome risk. Fertil Steril.

[10] El-Shal AS, Shalaby SM, Aly NM, Rashad NM, Abdelaziz AM (2013). Genetic variation in the vitamin D receptor gene and vitamin D serum levels in Egyptian women with polycystic ovary syndrome. Mol Biol Rep.

[11] Mahmoudi T, Majidzadeh-A K, Farahani H, Mirakhorli M, Dabiri R, Nobakht H (2015). Association of vitamin D receptor gene variants with polycystic ovary syndrome: a case control study. Int J Reprod Biomed (Yazd).

[12] Siddamalla S, Reddy TV, Govatati S, Erram N, Deenadayal M, Shivaji S (2017). Vitamin D receptor gene polymorphisms and risk of polycystic ovary syndrome in south Indian women. Gynecol Endocrinol.

[13] Wehr E, Trummer O, Giuliani A, Gruber HJ, Pieber TR, Obermayer-Pietsch B (2011). Vitamin D-associated polymorphisms are related to insulin resistance and vitamin D deficiency in polycystic ovary syndrome. Eur J Endocrinol.

[14] Dasgupta S, Dutta J, Annamaneni S, Kudugunti N, Battini MR (2015). Association of vitamin D receptor gene polymorphisms with polycystic ovary syndrome among Indian women. Indian J Med Res.

[15] Jedrzejuk D, Laczmanski L, Milewicz A, Kuliczkowska-Plaksej J, Lenarcik-Kabza A, Hirnle L (2015). Classic PCOS phenotype is not associated with deficiency of endogenous vitamin D and VDR genepolymorphisms rs731236 (TaqI), rs7975232 (ApaI), rs1544410 (BsmI), rs10735810 (FokI): a case-control study of lower Silesian women. Gynecol Endocrinol.

[16] Al-Daghri NM, Al-Attas OS, Alkharfy KM, Khan N, Mohammed AK, Vinodson B (2014). Association of VDR-gene variants with factors related to the metabolic syndrome, type 2 diabetes and vitamin D deficiency. Gene.

[17] Hitman GA, Mannan N, McDermott MF, Aganna E, Ogunkolade BW, Hales CN (1998). Vitamin D receptor gene polymorphisms influence insulin secretion in Bangladeshi Asians. Diabetes.

[18] Bienertová-Vašků J, Zlámal F, Pohořalá A, Mikeš O, Goldbergová-Pávková M, Novák J, Šplíchal Z, Pikhart H. Allelic variants in vitamin D receptor gene are associated with adiposity measures in the central-European population. BMC Medical Genetics. 2017;18:90–99. 10.1186/s12881-017-0454-z PMC5568207 28830368

[19] Alvarez JA, Ashraf A (2010). Role of vitamin D in insulin secretion and insulin sensitivity for glucose homeostasis. Int J Endocrinol.

[20] Oh JY, Barrett-Connor E (2002). Association between vitamin D receptor polymorphism and type 2 diabetes or metabolic syndrome in community-dwelling older adults: the rancho Bernardo study. Metabolism.

[21] Ortlepp JR, Metrikat J, Albrecht M, von Korff A, Hanrath P, Hoffmann R (2003). The vitamin D receptor gene variant and physical activity predicts fasting glucose levels in healthy young men. Diabet Med.

[22] Santos BR, Mascarenhas LPG, Satler F, Boguszewski MCS, Spritzer PM (2012). Vitamin D deficiency in girls from South Brazil: a cross-sectional study on prevalence and association with vitamin D receptor gene variants. BMC Pediatr.

[23] Han FF, Lv YL, Gong LL, Liu H, Wan ZR, Liu LH (2017). VDR gene variation and insulin resistance related diseases. Lipids Health Dis.

[24] Santos BR, Lecke SB, Spritzer PM (2017). Genetic variant in vitamin D-binding protein is associated with metabolic syndrome and lower 25-hydroxyvitamin D levels in polycystic ovary syndrome: a cross-sectional study. PLoS One.

[25] Group REA-SPCW (2004). Revised 2003 consensus on diagnostic criteria and long-term health risks related to polycystic ovary syndrome. Fertil Steril.

[26] Toscani M, Mighavacca R, Sisson de Castro JA, Spritzer PM (2007). Estimation of truncal adiposity using waist circumference or the sum of trunk skinfolds: a pilot study for insulin resistance screening in hirsute patients with or without polycystic ovary syndrome. Metabolism.

[27] Graff SK, Mario FM, Alves BC, Spritzer PM (2013). Dietary glycemic index is associated with less favorable anthropometric and metabolic profiles in polycystic ovary syndrome women with different phenotypes. Fertil Steril.

[28] Di Domenico K, Wiltgen D, Nickel FJ, Magalhaes JA, Moraes RS, Spritzer PM (2013). Cardiac autonomic modulation in polycystic ovary syndrome: does the phenotype matter?. Fertil Steril.

[29] Ramos RB, Spritzer PMFTO (2015). Gene variants are not associated with polycystic ovary syndrome in women from southern Brazil. Gene.

[30] Alberti K, Eckel RH, Grundy SM, Zimmet PZ, Cleeman JI, Donato KA (2009). Harmonizing the metabolic syndrome a joint interim statement of the international diabetes federation task force on epidemiology and prevention; National Heart, Lung, and Blood Institute; American Heart Association; world heart federation; international atherosclerosis society; and International Association for the Study of obesity. Circulation.

[31] Wallace TM, Levy JC, Matthews DR (2004). Use and abuse of HOMA modeling. Diabetes Care.

[32] Miller SA, Dykes DD, Polesky HF (1988). A simple salting out procedure for extracting DNA from human nucleated cells. Nucleic Acids Res.

[33] La Marra F, Stinco G, Buligan C, Chiriacò G, Serraino D, Di Loreto C (2017). Immunohistochemical evaluation of vitamin D receptor (VDR) expression in cutaneous melanoma tissues and four VDR gene polymorphisms. Cancer Biol Med.

[34] Ogunkolade BW, Boucher BJ, Prahl JM, Bustin SA, Burrin JM, Noonan K (2002). Vitamin D receptor (VDR) mRNA and VDR protein levels in relation to vitamin D status, insulin secretory capacity, and VDR genotype in Bangladeshi Asians. Diabetes.

[35] Meyer V, Saccone DS, Tugizimana F, Asani FF, Jeffery TJ, Bornman L (2017). Methylation of the vitamin D receptor (VDR) gene, together with genetic variation, race, and environment influence the signaling efficacy of the toll-like receptor 2/1-VDR pathway. Front Immunol.

[36] Uitterlinden AG, Fang Y, Van Meurs JB, Pols HA, Van Leeuwen JP (2004). Genetics and biology of vitamin D receptor polymorphisms. Gene.

[37] Pike JW, Meyer MB (2010). The vitamin D receptor: new paradigms for the regulation of gene expression by 1,25-Dihydroxyvitamin D-3. Endocrinol Metab Clin N Am.

[38] Dilworth FJ, Chambon P (2001). Nuclear receptors coordinate the activities of chromatin remodeling complexes and coactivators to facilitate initiation of transcription. Oncogene.

[39] Joham AE, Teede HJ, Cassar S, Stepto NK, Strauss BJ, Harrison CL (2016). Vitamin D in polycystic ovary syndrome: relationship to obesity and insulin resistance. Mol Nutr Food Res.

[40] Bahrami A, Sadeghnia HR, Tabatabaeizadeh SA, Bahrami-Taghanaki H, Behboodi N, Esmaeili H, Ferns GA, Mobarhan MG, Avan A (2018). Genetic and epigenetic factors influencing vitamin D status. J Cell Physiol.

[41] Bagheri M, Abdi Rad I, Hosseini Jazani N, Nanbakhsh F (2013). Vitamin D receptor TaqI gene variant in exon 9 and polycystic ovary syndrome risk. Int J Fertil Steril.

[42] San-Pedro JI, Bilbao JR, De Nanclares GP, Vitoria JC, Martul P, Castano L (2005). Heterogeneity of vitamin D receptor gene association with celiac disease and type 1 diabetes mellitus. Autoimmunity.

[43] Ramos-Lopez E, Jansen T, Ivaskevicius V, Kahles H, Klepzig C, Oldenburg J (2006). Protection from type 1 diabetes by vitamin D receptor haplotypes. Ann N Y Acad Sci.

[44] Panierakis C, Goulielmos G, Mamoulakis D, Petraki E, Papavasiliou E, Galanakis E (2009). Vitamin D receptor gene polymorphisms and susceptibility to type 1 diabetes in Crete, Greece. Clin Immunol.

[45] Kamel MM, Fouad SA, Salaheldin O, Abd El-Razek AA, Abd El-Fatah AI (2014). Impact of vitamin D receptor gene polymorphisms in pathogenesis of Type-1 diabetes mellitus. Int J Clin Exp Med.

